# Large Vasoproliferative Retinal Tumor Refractory to Cryotherapy Treated with Salvage I-125 Plaque Radiation Therapy

**DOI:** 10.1016/j.adro.2022.100972

**Published:** 2022-04-22

**Authors:** Matthew J. Case, Connor Lentz, Edward William Duffy, George Nathani Magrath, Samuel Lewis Cooper

**Affiliations:** a College of Medicine, Medical University of South Carolina, Charleston, South Carolina; b Department of Radiation Oncology, Medical University of South Carolina, Charleston, South Carolina; c Department of Ophthalmology, Medical University of South Carolina, Charleston, South Carolina

## Introduction

Vasoproliferative retinal tumors (VPRTs) were initially described through case reports as early as 1966 as “angioma-like” lesions arising in the retina in the setting of patients with various other primary conditions including Coat's disease, Sickle cell disease, and retrolental fibroplasia.^1^, ^2^, ^3^, ^4^ These lesions were noted to be distinct from the hemangiomas seen in Von Hippel Lindau disease owing to the lack of genetic inheritance and possessing a less noticeably tortuous system of vessels.^1^ In the 1980s, a case series elucidated a pattern of disease often involving an inferotemporal location, an association with concurrent macula edema, and frequently arising in the setting of chronic inflammation.^5^, ^6^, ^7^ In a 1995 larger series of 103 patients by Shields et al, the term “vasoproliferative retinal tumor” was coined to describe these presumed acquired retinal hemangiomas.^1^^,^^8^ They were believed to arise secondary to some primary process, although oftentimes a primary process was not identified, and they were labeled as idiopathic in origin. Despite its name, VPRTs were eventually found to be composed primarily of glial cell proliferation with a leaky blood supply.^9^ Poole Perry et al have suggested a more appropriate name of “retinal reactive astrocytic tumor.”^9^

Although the tumors themselves are often benign, the lesions can cause secondary complications.^10^ Given their leaky vasculature, retinal exudation with retinal detachment may occur. Patients frequently present with poor visual acuity, floaters, visual distortion, photopsia, or even blindness.^10^ To preserve vision, close observation or treatment is warranted. The decision to treat or not is dependent on symptoms, tumor size, and other features on eye examination.^11^ A uniform staging system to guide treatment is not well accepted, though recently one system was described by Honavar.^12^ Tumors with greater potential for vision loss based on the extent of secondary complications were staged higher and warranted greater consideration of treatment.^12^

A variety of treatment options exist which include cryotherapy, diathermy, antiangiogenic therapy, immunosuppression, anti-inflammatory treatment, resection, photodynamic therapy (PDT), laser therapy, and plaque radiation therapy.^11^ The efficacy of each treatment option has been documented largely through case reports and series. Several studies have highlighted the importance of tumor size, location, secondary complications, hospital resources, and patient preference in deciding management.^11^^,^^13^, ^14^ To date, no randomized controlled trials have been performed to compare the efficacy of varying treatments.

Cryotherapy has historically been the preferred treatment option and has shown excellent control of disease. In the 1995 retrospective study by Shields et al of 103 patients, 49% of all cases were managed with close observation followed by 42% treated with cryotherapy, 5% treated with PDT, 2% treated with plaque radiation therapy, and 2% treated by other modalities.^8^ Of the 23 patients classified as having secondary VPRTs, the largest VPRT basal diameter measured less than 16 mm, and only one VPRT had a thickness greater than 6 mm. A 2014 retrospective study of 16 patients treated with cryotherapy with 68 months of follow-up showed 100% tumor response rate.^15^ In this study, average tumor base diameter measured 6 mm with an average thickness of 3 mm. For larger tumors with diameter greater than 10 mm or thickness greater than 2.5 mm, cryotherapy is often avoided due to the risk of causing increased subretinal exudation or hemorrhage.^16^^,^^17^ Similarly, other locally ablative therapies such as PDT and laser therapy have proven effective in treating smaller, accessible VPRTs but become more technically challenging for tumors involving the periphery of the retina.^18^^,^^19^

Plaque radiation therapy was shown to be effective in the early 2000s and has since gained popularity, particularly for larger tumors. Treatment with either Ru-106 or I-125 sources allow for excellent tumor coverage while minimizing treatment complications. I-125 in particular emits gamma radiation and allows for greater penetration of tissue. Ru-106 decays via beta emission which has less penetration depth but allows for greater sparing of normal tissue structures. A 2006 retrospective study of 35 patients treated with Ru-106 brachytherapy showed disease control in 88.6% of patients.^18^ A separate study in 2020 of 25 patients showed disease response in 100% of patients.^20^ Most relevant to this case, a 2008 retrospective study of 30 patients treated with I-125 plaque radiation therapy showed tumor response in 97% of patients when treating to an apex dose of 40 Gy.^21^ Notably, tumor thickness averaged 3.7 mm and ranged from 2.5 to 6.3 mm, and basal diameter averaged 8.6 mm and ranged from 3.5 to 18.0 mm. Herein, we describe a VPRT with a 7 mm thickness and 20 mm diameter refractory to cryotherapy and successfully treated with salvage I-125 plaque radiation therapy. Institutional Review Board approval was not required as a case report of 3 or fewer patients was not considered human-subject research by the institution.

## Case Presentation

A 23-year-old woman with bilateral VPRTs was referred for management. She had a reported history of difficulty with vision starting at age 15. At age 18, the patient received a diagnosis of right eye pars planitis and neovascular glaucoma. At age 19, the first VPRT was noted in the patient's right eye. This was initially treated at an outside hospital with cryotherapy. At age 23, the patient was subsequently noted to have bilateral pars planitis with VPRTs. She was otherwise healthy with no family history of eye disease.

**Fig. 1:**
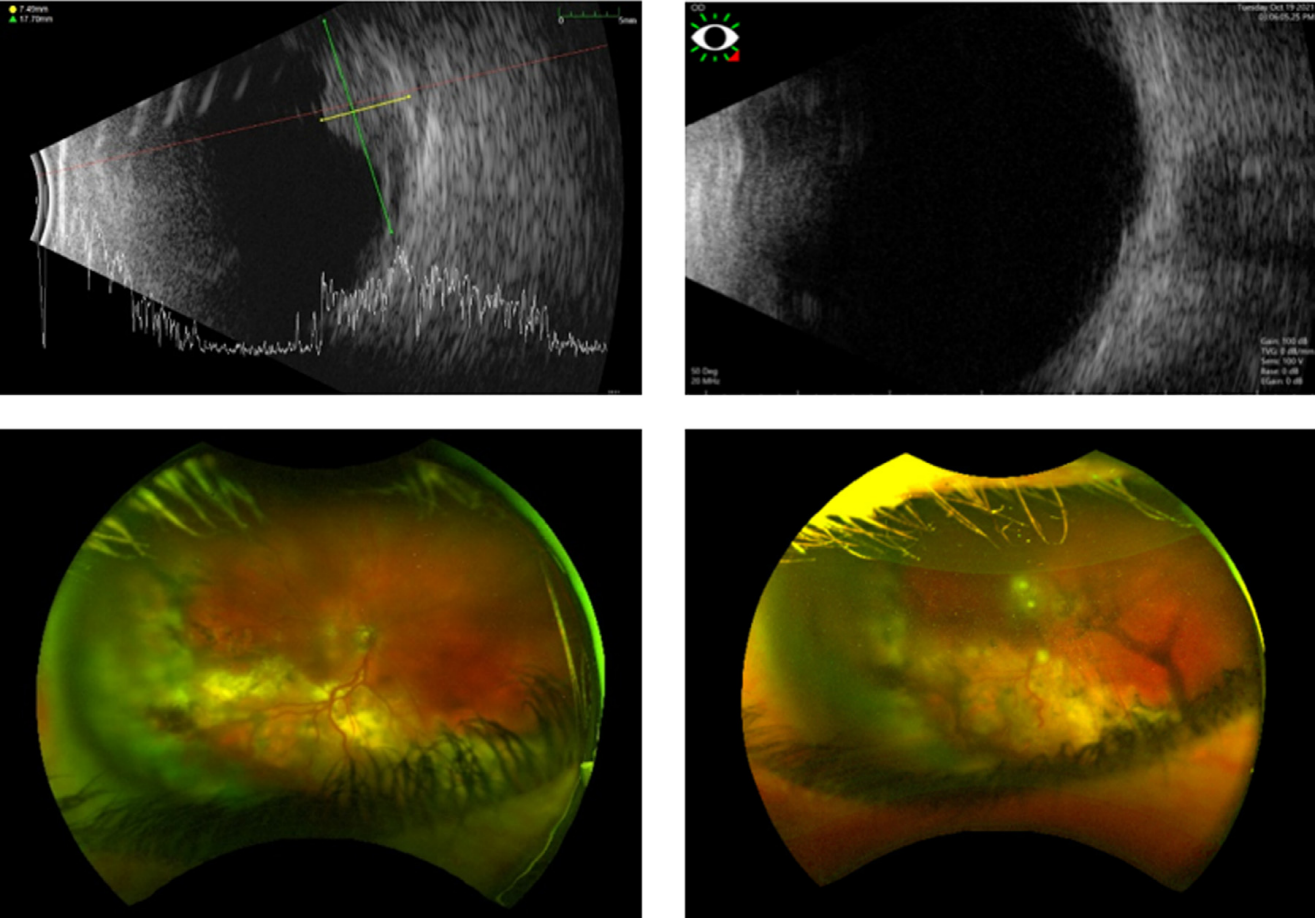
Pretreatment B-scan ultrasound and fundus image findings of right eye (left side) and 7 months after radiation therapy follow-up imaging (right side).

Visual acuity was measured at 20/100 in the right eye and 20/20 in the left eye. The right eye had diffuse constriction on Humphrey visual field testing. IOP was 29 mm Hg in the right eye and 16 mm Hg in the left. Fundus examination revealed bilateral VPRTs inferiorly with tortuous vessels (Fig 1). The right lesion was approximately 7 mm thick with a 20 mm basal diameter with significant inferior exudate and associated retinal detachment. The right lesion carried a significantly guarded prognosis and was classified as a stage 5a tumor on account of the exudative retinal detachment and neovascular glaucoma based on Honavar's staging. The left lesion was 2 mm thick and classified as stage 3a due to epiretinal membrane involving the fovea.

Management options were discussed with the patient which included treatment versus observation. Treatment with laser therapy, cryotherapy, and antiangiogenic therapy were felt to be unlikely to control a tumor of the size found in the right eye. Consequently, plaque radiation therapy was preferred for the right eye on account of its size and associated retinal detachment, and the smaller left eye lesion was planned for cryotherapy at a later date.

**Fig. 2:**
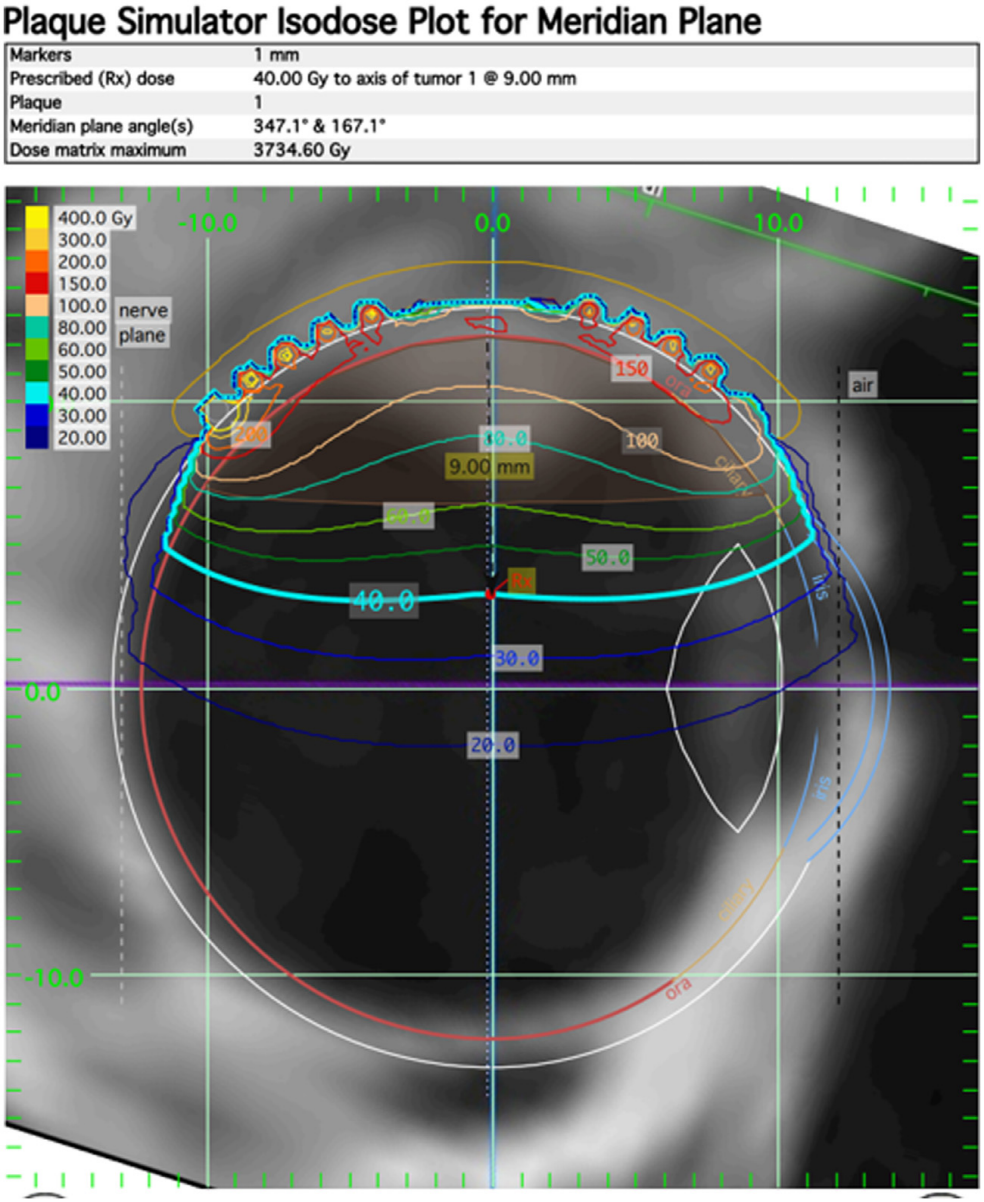
Placement and isodose lines of I-125 plaque radiation therapy treatment onto the right eye vasoproliferative retinal tumor.

**Fig. 3:**
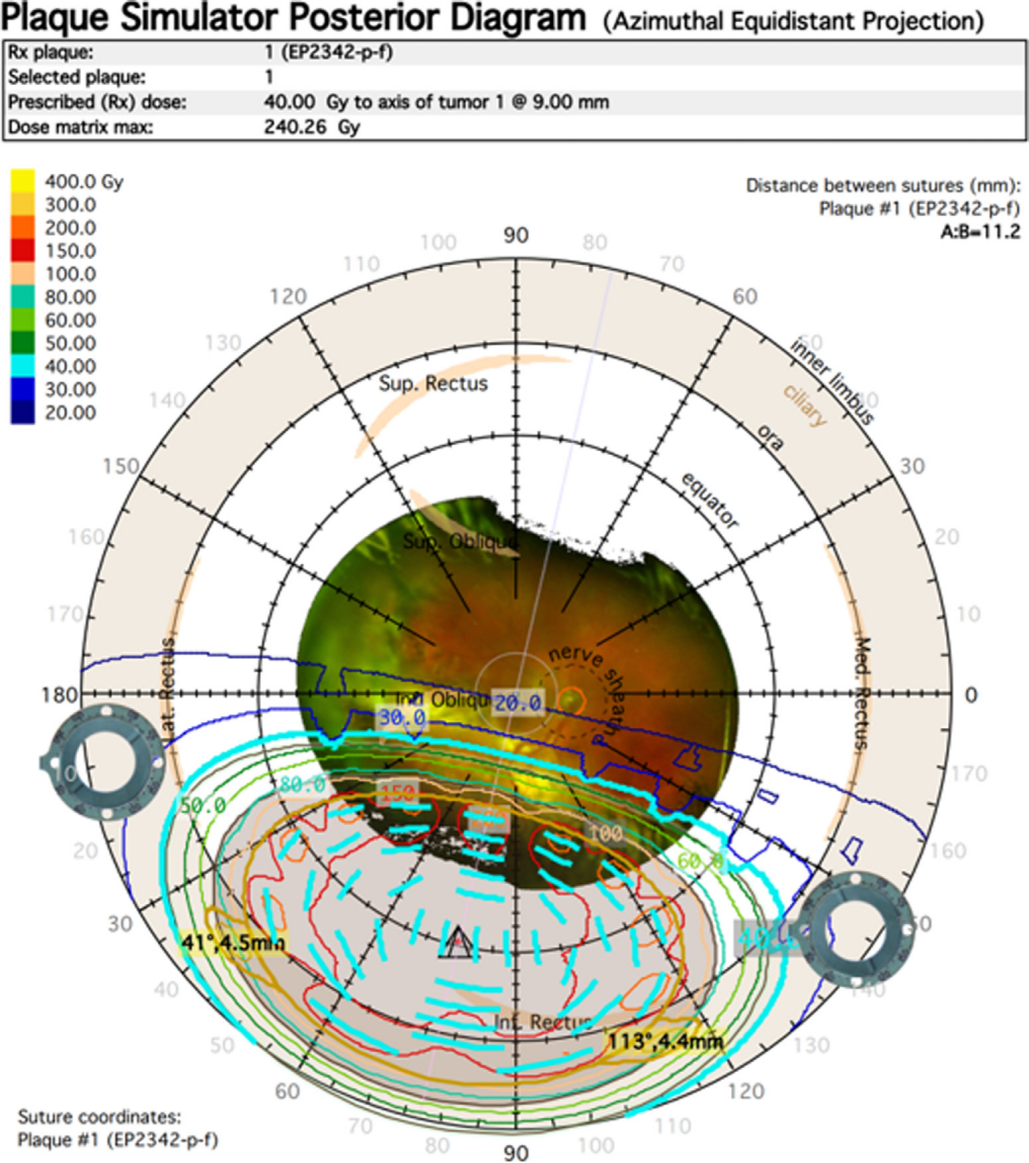
Plaque simulator posterior diagram (azimuthal equidistant projection).

The right eye lesion was treated with the Eye Physics I-125 plaque radiation therapy model EP2342-*P*-f with 42 seeds with a target dose of 40 Gy to a depth of 9 mm. The prescription depth was chosen to adequately cover the 20 mm basal diameter and resulted in a dose of 60 Gy to the apex of the tumor. The dose to the disc center was 17.6 Gy and the dose to the fovea was 19.3 Gy (Fig. 2 and 3). On day 0 of the procedure, the patient was placed under general and regional anesthesia in the OR and the plaque was surgically placed. On day 4 of the procedure, the plaque was removed. The patient tolerated the procedure well with minimal eye pain and itching managed with medications.

At 1-month follow-up, the exudative reaction had decreased compared with before the operation and visual acuity had improved to 20/80 in the right eye. Glaucoma persisted in the right eye and was medically managed. At 3-months follow-up**,** the exudative reaction was again noted to be decreased and visual acuity was similar at 20/100 in the right eye. At 5-months follow-up, the persistent right eye macular edema was treated with intravitreal dexamethasone injection (Ozurdex). At 6-months follow-up, the exudative reaction had again decreased compared with before the operation and visual acuity was improved to 20/60 in the right eye. Macular edema showed significant improvement with Ozurdex. B-scan image taken at 7-months showed significant tumor regression from before treatment (Fig. 1).

## Discussion

In this case report, we described successful salvage treatment of a particularly large VPRT that initially arose possibly secondary to chronic pars planitis. The lesion was refractory to cryotherapy and then subsequently controlled with I-125 plaque radiation therapy as of 8 months. The case was notable for the size of the VPRT and its initial resistance to cryotherapy.

Salvage plaque radiation therapy treatment of VPRTs after initial cryotherapy has been infrequently described. The largest case series to date of VPRTs treated with I-125 included 2 of 30 who were treated after failed cryotherapy.^21^ Although a subset of these patients was not included, the cohort was treated to a mean apex dose of 40 Gy (range, 20-90 Gy). A separate retrospective study of 38 patients treated with Ru-106 plaque radiation therapy to a mean apex dose of 90 Gy included 4 of 38 patients which had initially failed cryotherapy.^22^ Although a subset analysis of these patients was not included, the authors found that larger tumors were more difficult to control, with tumors resistant to therapy having an average diameter of 7.9 mm compared with 5.9 mm in tumors responding to therapy (*P* = .0007). It should be noted that while the Ru-106 decay mechanism allows for higher apex dose while sparing normal tissues, treatment may be more susceptible to set-up error due to the steep dose gradient. In the case described herein, the tumor measured 20 mm in basal diameter and was successfully controlled with I-125 to apex dose of only 60 Gy.

Although treatment of large VPRTs with thickness >2.5 mm or basal diameter >10 mm are often successfully treated with plaque radiation therapy alone, there may be certain cases where upfront combination therapy is preferred. In the previous study, 3 of 38 patients initially treated with plaque radiation therapy subsequently required salvage cryotherapy for disease control. The authors concluded that planned combination cryotherapy and plaque radiation therapy may be suitable for larger VPRTs. A 2020 retrospective Chinese study of 20 patients included 16 patients who were treated with various combinations of Ru-106 plaque radiation therapy, PDT, anti-VEGF therapy, and cryotherapy.^23^ Of these 16 patients, 9 of 16 showed decreased complications and improved visual acuity compared with monotherapy, 3 of 16 showed no difference, and 2 of 16 showed worsened complications and visual acuity.^23^ The study was limited by poor sample size in the monotherapy group. Future studies regarding upfront combination treatment, which also include I-125 plaque radiation therapy are warranted.

## Conclusions

VPRTs are a benign reactionary condition histologically distinct from the retinal hemangiomas seen in Von Hippel Lindau disease. Lesions are typically well controlled with either cryotherapy or radiation therapy, although a variety of other modalities have been reported. To date, no randomized controlled trials have been performed to compare treatment options. Data regarding treatment in the salvage setting is particularly limited. In this case report, we described a patient with bilateral VPRTs likely secondary to chronic pars planitis. The right eye lesion was particularly large and initially refractory to cryotherapy and then successfully treated with salvage I-125 plaque radiation therapy.

## Appendix Supplementary materials

Image, application 1
