# Supplementary material for: Large Vasoproliferative Retinal Tumor Refractory to Cryotherapy Treated with Salvage I-125 Plaque Radiation Therapy
Source: Adv Radiat Oncol. 2022 Apr 22;7(5):100972. doi: 10.1016/j.adro.2022.100972 (PMC9160674; doi:10.1016/j.adro.2022.100972)
Supplement: Supplementary file 1 [file mmc1.pdf]

Supplemental Figure 1: Right eye OCT and fundus imaging at 1 month pretreatment with I-125 plaque radiotherapy (furthest left), and then at 1 months, 3 months, and 6 months post-treatment with I-125 plaque radiotherapy (furthest right).

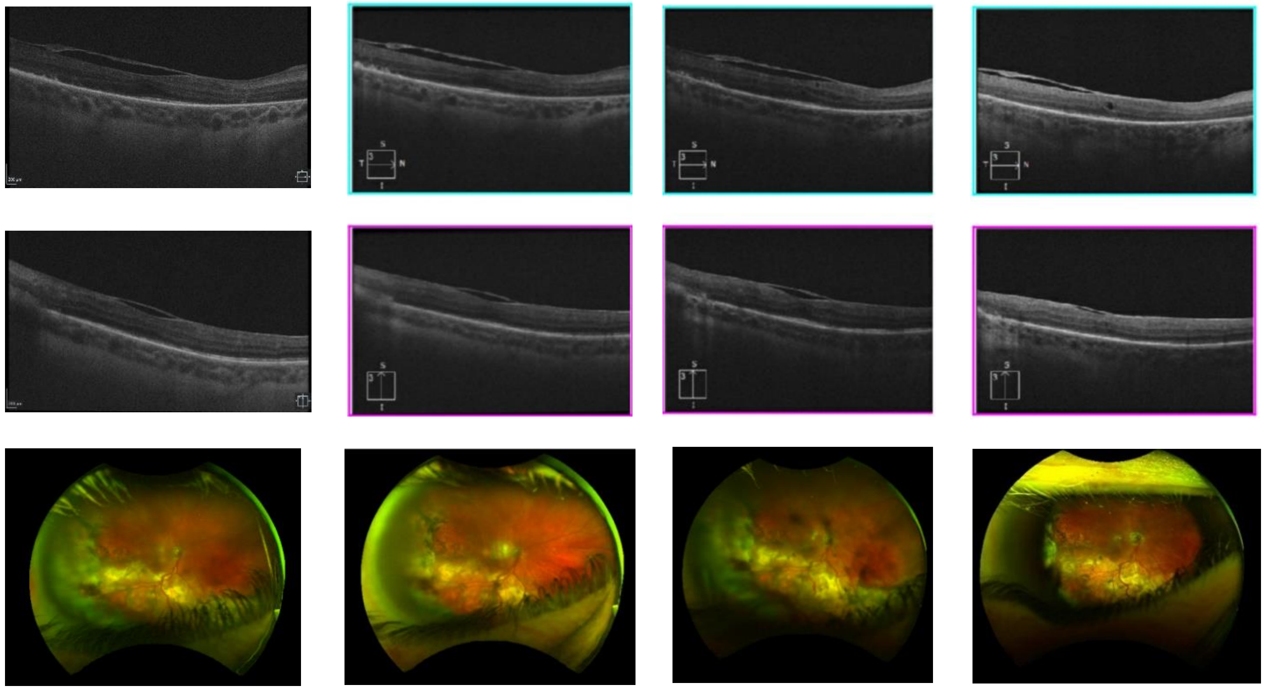

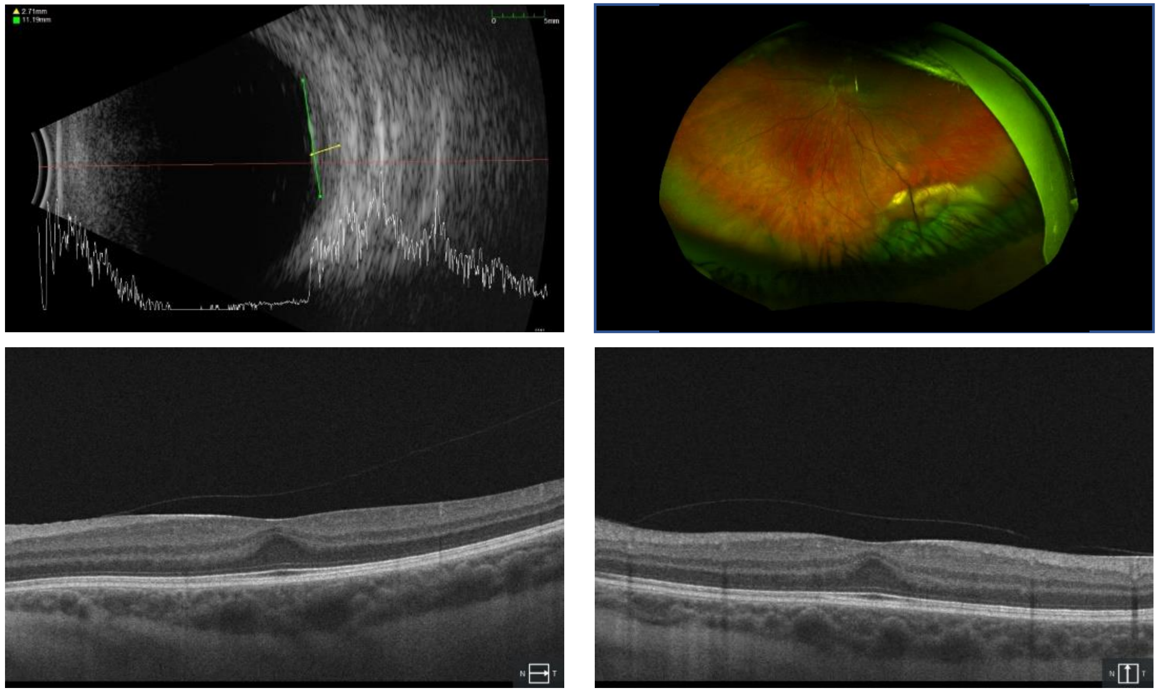

Supplemental Figure 2: Presenting OCT and fundus image findings of left eye with small VPRT noted that would eventually be treated with cryotherapy.
